# Electrical muscle stimulation preserves the muscle mass of critically ill patients: a randomized study

**DOI:** 10.1186/cc8123

**Published:** 2009-10-08

**Authors:** Vasiliki Gerovasili, Konstantinos Stefanidis, Konstantinos Vitzilaios, Eleftherios Karatzanos, Panagiotis Politis, Apostolos Koroneos, Aikaterini Chatzimichail, Christina Routsi, Charis Roussos, Serafim Nanas

**Affiliations:** 1 First Critical Care Department, Evangelismos Hospital, National and Kapodistrian University of Athens, 45-47 Ypsilantou Str., 106 75, Athens, Greece; 2 Second Department of Radiology, Attiko Hospital, National and Kapodistrian University of Athens, 1 Rimini Str.,12462, Athens, Greece

## Abstract

**Introduction:**

Critically ill patients are characterized by increased loss of muscle mass, partially attributed to sepsis and multiple organ failure, as well as immobilization. Recent studies have shown that electrical muscle stimulation (EMS) may be an alternative to active exercise in chronic obstructive pulmonary disease (COPD) and chronic heart failure (CHF) patients with myopathy. The aim of our study was to investigate the EMS effects on muscle mass preservation of critically ill patients with the use of ultrasonography (US).

**Methods:**

Forty-nine critically ill patients (age: 59 ± 21 years) with an APACHE II admission score ≥13 were randomly assigned after stratification upon admission to receive daily EMS sessions of both lower extremities (EMS-group) or to the control group (control group). Muscle mass was evaluated with US, by measuring the cross sectional diameter (CSD) of the vastus intermedius and the rectus femoris of the quadriceps muscle.

**Results:**

Twenty-six patients were finally evaluated. Right rectus femoris and right vastus intermedius CSD decreased in both groups (EMS group: from 1.42 ± 0.48 to 1.31 ± 0.45 cm, *P *= 0.001 control group: from 1.59 ± 0.53 to 1.37 ± 0.5 cm, *P *= 0.002; EMS group: from 0.91 ± 0.39 to 0.81 ± 0.38 cm, *P *= 0.001 control group: from 1.40 ± 0.64 to 1.11 ± 0.56 cm, *P *= 0.004, respectively). However, the CSD of the right rectus femoris decreased significantly less in the EMS group (-0.11 ± 0.06 cm, -8 ± 3.9%) as compared to the control group (-0.21 ± 0.10 cm, -13.9 ± 6.4%; *P *< 0.05) and the CSD of the right vastus intermedius decreased significantly less in the EMS group (-0.10 ± 0.05 cm, -12.5 ± 7.4%) as compared to the control group (-0.29 ± 0.28 cm, -21.5 ± 15.3%; *P *< 0.05).

**Conclusions:**

EMS is well tolerated and seems to preserve the muscle mass of critically ill patients. The potential use of EMS as a preventive and rehabilitation tool in ICU patients with polyneuromyopathy needs to be further investigated.

**Trial Registration:**

clinicaltrials.gov: NCT00882830

## Introduction

Critical illness polyneuromyopathy (CIPNM) is a common complication of critical illness presenting with muscle weakness, diminished tendon reflexes, difficult weaning from mechanical ventilation [1,2], and prolonged intensive care unit (ICU) and hospital stay [2,3], and is associated with increased mortality [4]. CIPNM is reported to have an incidence ranging from 25 to 50% [5,6] or higher [7] depending on the criteria used for diagnosis and the patient population evaluated. In afflicted patients muscle weakness may persist for months and a percentage may never fully recover [8]. Intensive insulin therapy has been proposed as a preventive therapy for CIPNM [9]. However, results from recent studies have raised concerns regarding the safety and the mortality benefit of intensive insulin therapy [10,11]. Despite its clinical significance, no preventive or therapeutic tool has been proposed so far for CIPNM.

Muscle mass is a component of function and improvements in the cross sectional area of a muscle have been shown to be associated with the increased strength and force in health [12] and disease [13]. Critically ill patients, especially those with CIPNM, are characterized by increased loss of muscle mass [14], partially attributed to sepsis and multiple organ failure, the use of drugs such as neuromuscular blocking agents, as well as immobilization. Sepsis is known to be a hypercatabolic state for the muscle [15]. Immobilization, even of short duration, is also a catabolic state for the muscle resulting in significant loss of muscle mass in healthy subjects [16], as well as in critically ill patients [17,18].

Electrical muscle stimulation (EMS) could be considered to be an alternative to active exercise, which does not require patient cooperation. EMS has been used in patients with severe chronic obstructive pulmonary disease (COPD) [19,20] and chronic heart failure (CHF) [21]. These patients cannot actively exercise due to cardiac and respiratory insufficiency, so they benefit from EMS in terms of exercise capacity [20-22], skeletal muscle performance [19,20,22] and quality of life [19,21]. EMS has been used in patients with severe COPD under mechanical ventilation [19] but it has not been used so far in critically ill ICU patients.

We hypothesized that EMS, as an alternative form of exercise, will prevent the loss of muscle mass of critically ill patients. The aim of our study was to assess the effect of EMS on muscle mass preservation in critically ill patients with the use of ultrasonography (US).

## Materials and methods

### Patients

All patients admitted to our multidisciplinary ICU during the study period were prospectively considered for inclusion in the study. Exclusion criteria were: age under 18 years, pregnancy, obesity (BMI >35 kg/m^2^), brain death, preexisting neuromuscular disease (e.g. myasthenia gravis), diseases with systemic vascular involvement such as lupus erythematosus, technical obstacles that did not allow the implementation of EMS such as bone fractures or skin lesions (e.g. skin burns) and end-stage malignancy. Patients with pacemakers and those with an ICU stay of less than 48 hours were also excluded from the study. The Sepsis Organ Failure Assessment (SOFA) [23], Acute Physiology and Chronic Health Evaluation (APACHE) II [24] and Simplified Acute Physiology Score (SAPS) 3 [25] severity scores were calculated for all patients on the day of admission. These scores have been developed for the assessment of disease severity and have prognostic value in patients admitted to the ICU. Patients with an APACHE II admission score of 13 or higher underwent, on the second day after admission, stratified randomization (age, gender) and were assigned to the intervention group (EMS group) or to the control group. Patients assigned to the EMS group received daily EMS sessions of both lower extremities (Table 1). Informed consent was obtained from patients or from the patients' relatives as approved by the Scientific Council and the Ethics Committee of Evangelismos Hospital.

**Table 1:** Baseline characteristics of critically ill patients randomly assigned to the EMS group or the control group (mean ± SD)

	EMS group	Control group	P
**Age (years)**	**59 ± 23**	**56 ± 19**	**NS**
**Gender (male/female)**	**6/7**	**8/5**	
**APACHE II admission**	**19 ± 3**	**18 ± 6**	**NS**
**SOFA admission**	**10 ± 3**	**8 ± 3**	**NS**
**SAPS 3 admission**	**66 ± 9**	**61 ± 14**	**NS**
**Mechanical ventilation (days, n)**	**9 ± 2 (4-10), 13**	**9 ± 3 (6-10), 12**	
**Reasons of ICU admission (n)**			
Sepsis	**5**	**5**	
Trauma	**4**	**3**	
Neurologic (including cerebrovascular)	**2**	**4**	
Other	**2**	**1**	
**Sedation (days, n)**	**5 ± 4, 9**	**6 ± 4, 11**	
**Vasopressors (days, n)**	**5 ± 4, 10**	**5 ± 4, 10**	
**Hyperglycemia (days, n)**	**4 ± 3, 11**	**4 ± 3,10**	
**Glucocorticoids (n)**	**4**	**4**	
**Neuromuscular blockers (n)**	**2**	**2**	
**Aminoglycosides (n)**	**6**	**7**	
**Sepsis developed (n)**	**9**	**10**	
**Hyperglycemia was defined as blood glucose level >140 mg/dl**			

Numbers in brackets signify range of values.

APACHE = Acute Physiology and Chronic Health Evaluation; EMS = electrical muscle stimulation; ICU = intensive care unit; NS = not significant; SAPS = Simplified Acute Physiology Score; SD = standard deviation; SOFA = Sequential Organ Failure Assessment.

### Electrical muscle stimulation session

EMS was implemented simultaneously on the quadriceps and peroneous longus muscles of both lower extremities daily from the second to ninth day after admission. In the case of hairy skin, the skin was carefully shaven before the application. After shaving and skin cleaning, rectangular electrodes (90 × 50 mm) were placed on the quadriceps and peroneus longus muscles of both legs. The stimulator (Rehab 4 Pro, CEFAR Medical AB, Malmö, Sweden) delivered biphasic, symmetric impulses of 45 Hz, 400 μsec pulse duration, 12 seconds on and 6 seconds off, at intensities able to cause visible contractions. In case of doubt, contraction was confirmed by palpation of the muscles involved. Mean intensities used were 38 ± 10 mA (range 19 to 55 mA) for quads and 37 ± 11 mA (range 23 to 60 mA) for peroneous longus. The duration of the session was 55 minutes including 5 minutes for warm up and 5 minutes for recovery. Sessions that took place were 81 ± 26%.

### Assessment of muscle mass

Muscle mass was evaluated with US, by measuring the Cross Sectional Diameter (CSD) of the quadriceps muscle (rectus femoris - vastus intermedius) [26-28] on the day of randomization (second day of admission) and seven or eight days after the first assessment [29]. The choice of the ultrasonographic measurement of quadriceps muscle was made, because it is the largest muscle of the whole body, is easily accessible and correlates well with lean tissue mass [30]. US images were obtained using a GE Vivid 7 model ultrasound scanner with a high frequency (7.5 MHz) linear transducer, appropriate for the superficial structures [31]. This technique has been shown to have very good intra- and inter-observer reproducibility [30]. The measurements were performed by two operators who were blinded to the randomization, while the repetition of the measurement was made by the same operator. All patients were in the supine position with legs lying flat in extension. The position of the probe was selected at the midway between the anterior superior iliac spine and the midpoint of the patella and was placed ventral to the transverse plane and perpendicular to bone surface. To prevent impression of the skin, an excess of gel was employed. To standardize the measurements, the position of the probe was marked for the following measurement.

### Statistical analysis

All continuous variables are presented by mean ± standard deviation. The within-patient changes were evaluated with Wilcoxon. The differences between patients were evaluated by nonparametric test (Mann-Whitney). The statistical significance of *P *value was set at 0.05.

## Results

**Figure 1:**
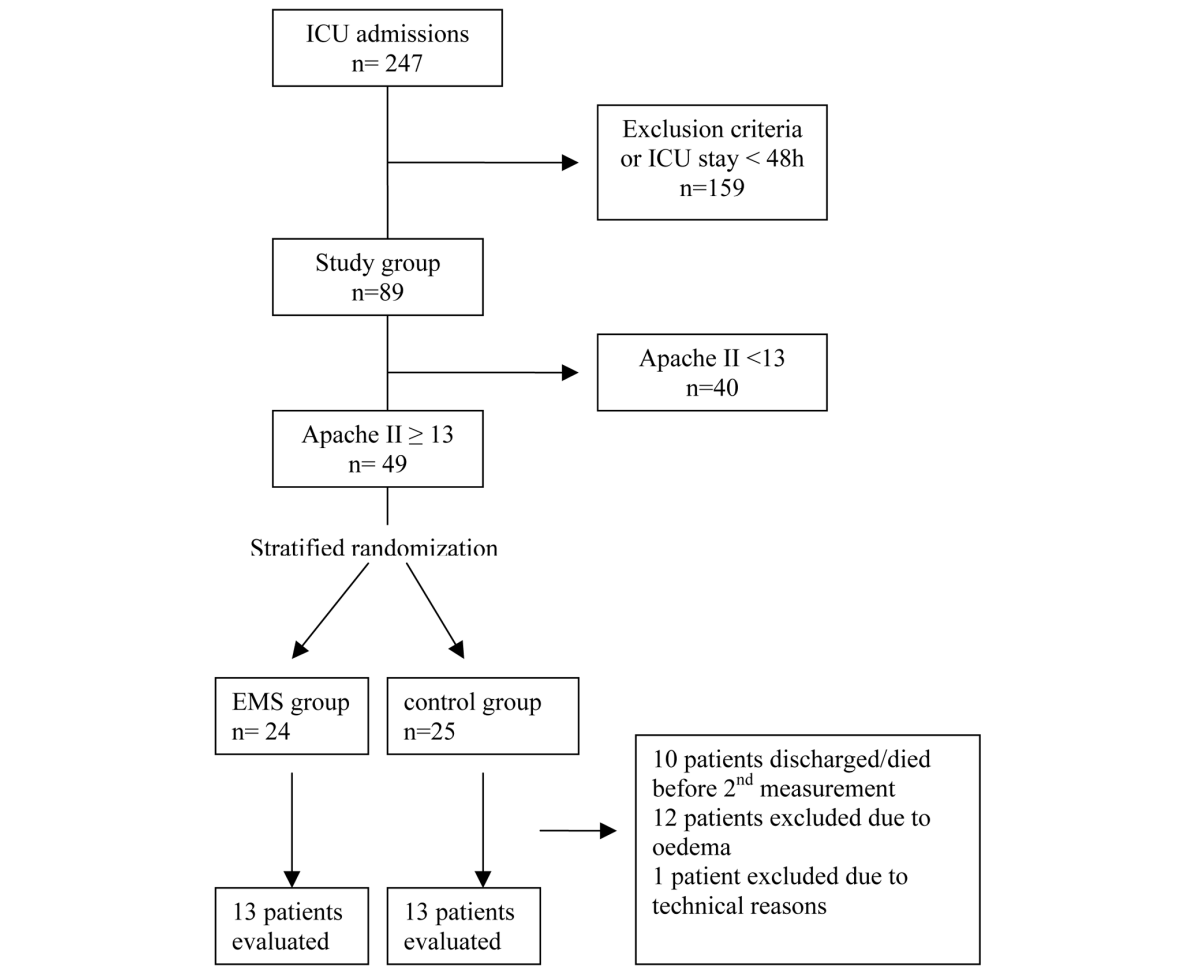
Schediagram of patients admitted to the ICU. APACHE = Acute Physiology and Chronic Health Evaluation; EMS = electrical muscle stimulation; ICU = intensive care unit.

Two hundred and forty-seven patients were admitted to our multidisciplinary ICU during the nine-month study period and 159 patients fulfilled the exclusion criteria or stayed in the ICU less than 48 hours. The study population consisted of 88 patients of which 49 patients had an APACHE II admission score of 13 or more. Of these patients, 24 were randomly assigned to the EMS group and 25 to the control group. Ten patients died or were discharged from the ICU before the second measurement, 12 patients were excluded due to oedema that interfered with the US measurements and 1 patient was not measured due to technical reasons. In the EMS group, 5 patients were excluded due to oedema and 6 patients died or were discharged before the second measurement. In the control group, 6 patients were excluded due to oedema, 5 patients died or were discharged before the second measurement and 1 patient could not be measured due to technical problems. Rectus femoris and vastus intermedius CSD could be assessed in 26 patients, 13 in the EMS group and 13 in the control group (Figure 1). In two patients the CSD of the left rectus femoris and vastus intermedius could not be assessed due to local oedema.

Baseline characteristics of patients randomly assigned to the EMS group or the control group are shown in Table 1. All patients were under mechanical ventilation (EMS group range 4 to 10 days, control group range 6 to 10 days) with the exception of one patient assigned to the control group, who was in need of respiratory support but was not mechanically ventilated (Table 1).

**Figure 2:**
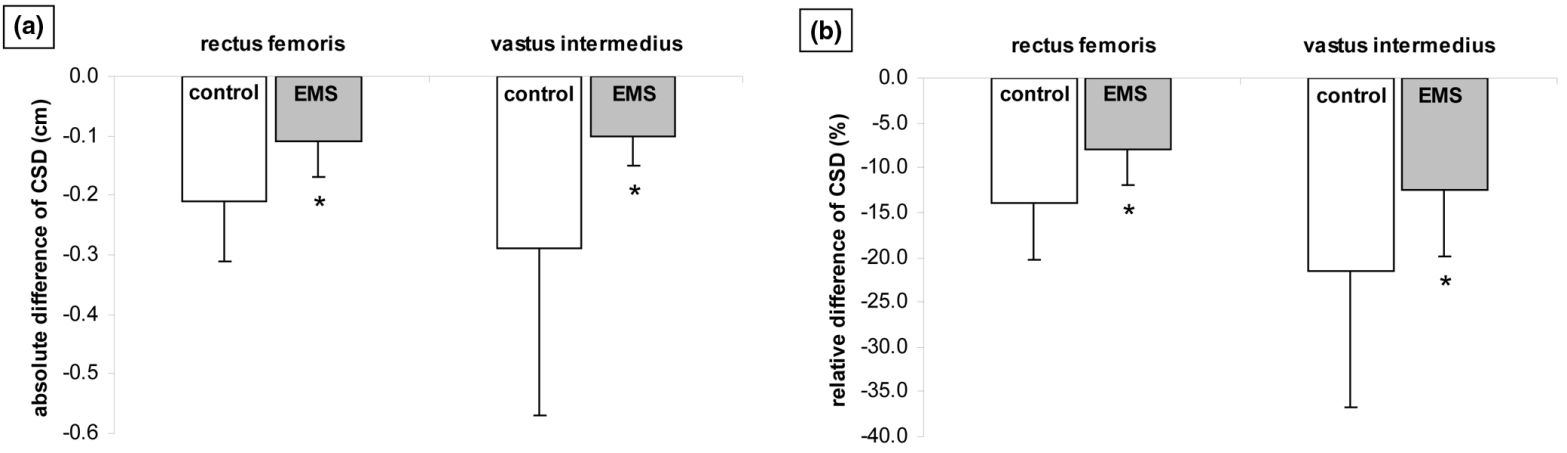
(a) Absolute difference (cm) and (b) relative difference (%) in cross sectional diameter (CSD) of right rectus femoris and vastus intermedius in the control (n = 13) and EMS (n = 13) groups (mean ± standard deviation). *significant between-group difference (*P *< 0.05). EMS = electrical muscle stimulation.

Right rectus femoris (EMS group: from 1.42 ± 0.48 to 1.31 ± 0.45 cm, *P *= 0.001; control group: from 1.59 ± 0.53 to 1.37 ± 0.5 cm, *P *= 0.002) and right vastus intermedius CSD (EMS group: from 0.91 ± 0.39 to 0.81 ± 0.38 cm, *P *= 0.001; control group: from 1.40 ± 0.64 to 1.11 ± 0.56 cm, *P *= 0.004) decreased significantly in both groups. However, the CSD of the right rectus femoris decreased significantly less in the EMS group (-0.11 ± 0.06 cm, -8 ± 3.9%) as compared with the control group (-0.21 ± 0.10 cm, -13.9 ± 6.4%; *P *= 0.009 for the absolute difference and *P *= 0.029 for the relative difference) and the CSD of the right vastus intermedius decreased significantly less in the EMS group (-0.10 ± 0.05 cm, -12.5 ± 7.4%) as compared with the control group (-0.29 ± 0.28 cm, -21.5 ± 15.3%; *P *= 0.034 for the absolute difference and *P *= 0.05 for the relative difference; Figure 2).

**Figure 3:**
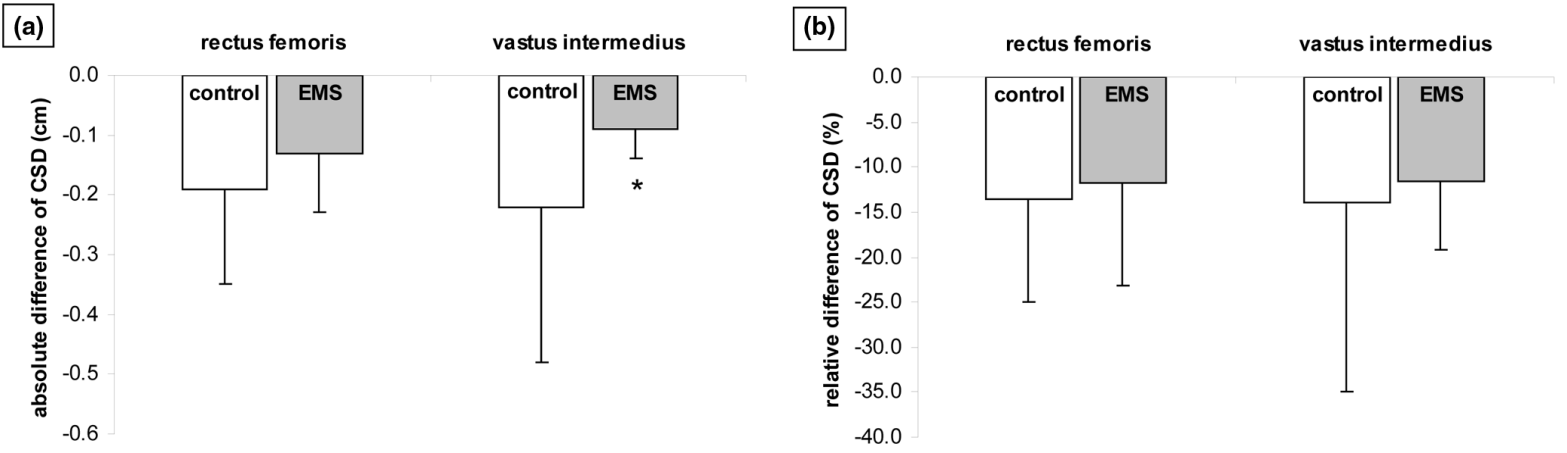
(a) Absolute difference (cm) and (b) relative difference (%) in cross sectional diameter (CSD) of left rectus femoris and vastus intermedius in the control (n = 13) and EMS (n = 13) groups (mean ± standard deviation). *significant between-group difference (*P *< 0.05). EMS = electrical muscle stimulation.

Left rectus femoris (EMS group: from 1.34 ± 0.39 to 1.2 ± 0.41 cm, *P *= 0.001; control group: from 1.62 ± 0.55 to 1.43 ± 0.6 cm, *P *= 0.014) and left vastus intermedius CSD (EMS group: from 0.86 ± 0.36 to 0.77 ± 0.35 cm, *P *= 0.001; control group: from 1.53 ± 0.67 to 1.31 ± 0.65 cm, *P *= 0.050) decreased significantly in both groups. However, the absolute difference in the CSD of the left rectus femoris was significantly less in the EMS group as compared with the control group (-0.13 ± 0.10 cm vs. -0.19 ± 0.16, *P *= 0.07) and the absolute difference in the CSD of the left vastus intermedius was significantly less in the EMS group as compared with the control group (-0.09 ± 0.05 cm vs -0.22 ± 0.26 cm, *P *= 0.018). The relative difference in the CSD of the left rectus femoris and left vastus intermedius was less in the EMS group as compared to the control group; however, the values did not reach statistical significance (-11.7 ± 11.5% vs -13.5 ± 11.5%, *P *= 0.331 and -11.6 ± 7.5% vs -14 ± 21%, *P *= 0.167, respectively; Figure 3).

## Discussion

The main finding of our randomized controlled study is that EMS of lower extremities seems to preserve the muscle mass of critically ill patients as assessed with US. To our knowledge, this is the first study to show that EMS of lower extremities applied to critically ill patients upon admission is associated with a lesser degree of muscle mass loss of these patients as assessed with US.

Critically ill patients undergo a state of hypermetabolism characterized by an increase in energy expenditure [32]. This condition is associated with increased protein loss, which to a large extent is attributed to skeletal muscle protein loss [18,32]. Moreover, immobilization even of short duration is known to have detrimental effects on skeletal muscle in healthy subjects [16] as well as in critically ill patients [14,18,19]. A recent case report showed that the loss of skeletal muscle mass may remain even after one year after ICU discharge despite an extensive rehabilitation program [33]. In our study, the muscle mass of critically ill patients as assessed with the CSD by US decreased in both groups; however, the decrease was significantly less in the intervention group. The CSD of the rectus femoris muscle decreased by 13.9% within one week in the control group. This severe loss of muscle mass during the first week of ICU stay is in accordance with that reported by other studies [14,17,32,33]. Therefore, a tool that could reverse this process and preserve the muscle structure and function, applied in the ICU setting would be desirable.

EMS has been used as an alternative to active exercise in patients with severe COPD [19,20] and CHF [21,22]. In these patients, EMS resulted in an improvement of muscle performance such as maximum voluntary contraction [20], muscle strength and endurance [34,35] but also resulted in structural changes of the muscle tissue [21]. In a recent study involving bed-bound patients with COPD receiving mechanical ventilation after ICU stay, EMS caused an increase in muscle strength and reduced the number of days for transfer from bed to chair [19]. Our study is the first to use EMS in critically ill patients in order to evaluate directly its effect on muscle mass preservation. However, in an early study, EMS was shown to have beneficial effects on muscle metabolism in ICU patients [36]. In our study, EMS during the first week of ICU stay preserved to a large extent the muscle mass of critically ill patients. In a study involving patients with spinal cord injury, three weeks of EMS increased the muscle thickness, as assessed with US, to near normal values [37]. EMS was well tolerated [38] and since it does not require the patient's cooperation it was easily applicable from the day of admission. EMS, as an alternative form of exercise, may act as an anabolic stimulus to the muscle reversing the catabolic effects of critical illness and immobilization.

The preservation of muscle mass was assessed with the use of US by measuring the CSD of two muscles, namely the rectus femoris and the vastus intermedius muscles. US is an easily applicable, non-invasive technique, which offers a cost-effective alternative for the measurement of muscle thickness [26,39]. Specifically, the thigh has been proposed for the assessment of muscle wasting in critically ill patients because it is well correlated with lean body mass [30].

### Clinical implication

This study aimed to assess the role of EMS for the preservation of muscle mass. Although the role of physical, occupational and mobility therapy has been increased in recent years [40,41], EMS is an alternative method of exercise causing minimal discomfort to patients who are not able to perform any form of physical exercise, as is often the case in critically ill patients. Functional evaluation and muscle strength would have been the most appropriate endpoints in our study. However, functional and muscle strength evaluation requires patient cooperation, which was not feasible for the majority of critically ill patients on the seventh or eighth day after admission. It is a limitation of this study that it did not evaluate the effect of EMS on the functional recovery or the muscle strength of critically ill patients, which would have been clinically significant endpoints. Further studies are needed to explore the possible role of EMS as a tool for preserving the muscle strength, the muscle properties and preventing CIPNM in critically ill patients and to define which patients would benefit most from this intervention.

### Limitations

Anticipated limitations were the presence of oedema and the extensive exclusion criteria that did not allow the evaluation of the muscle mass in a considerable number of patients in both legs of the study. Measurements can be confounded by oedema. Oedema also distorts US images and does not allow us to delimit rectus femoris and vastus intermedius. For these reasons, patients with oedema were not measured. However, the number of patients excluded due to oedema and early death or discharge was equally distributed between the intervention group and the control group.

Another limitation was the relatively small number of critically ill patients that were evaluated, which is under power for definite conclusions. Finally, no data as to functional recovery of the patients are reported in this study.

## Conclusions

EMS is well tolerated and seems to preserve the muscle mass of critically ill patients. Oedema has limited our conclusions significantly and as a result our conclusions can only apply to patients who do not develop oedema during their ICU stay. Whether EMS can also preserve muscle structure and function and eventually prevent CIPNM in critically ill patients needs to be further explored.

## Key messages

• CIPNM is a common complication of critical illness, for which no preventive or therapeutic tool has been reported so far

• EMS is well tolerated and seems to preserve the muscle mass of critically ill patients

• Further studies are needed to evaluate whether EMS can also preserve muscle structure and function and eventually prevent CIPNM

## Abbreviations

APACHE: Acute Physiology and Chronic Health Evaluation; CHF: chronic heart failure; CIPNM: critical illness polyneuromyopathy; COPD: chronic obstructive pulmonary disease; CSD: cross sectional diameter; EMS: electrical muscle stimulation; ICU: intensive care unit; SAPS: Simplified Acute Physiology Score; SOFA: Sequential Organ Failure Assessment; US: ultrasonography.

## Competing interests

The authors declare that they have no competing interests.

## Authors' contributions

All authors have contributed substantially to the submitted work and have read and approved the final manuscript. In particular VG participated in the design of the study, data acquisition, analysis and drafting of the manuscript. KS, KV and LK participated in data acquisition, analysis and drafting of the manuscript. PP, AK and AC revised critically the manuscript. CR helped with data analysis, revised critically the manuscript and gave approval for submission. CR revised critically the manuscript and gave the approval for submission. Finally, SN conceived of and helped with the coordination of the study, revised critically the manuscript and provided final approval.
